# Productivity of Malaria Vectors from Different Habitat Types in the Western Kenya Highlands

**DOI:** 10.1371/journal.pone.0019473

**Published:** 2011-04-29

**Authors:** Bryson A. Ndenga, Jemimah A. Simbauni, Jenard P. Mbugi, Andrew K. Githeko, Ulrike Fillinger

**Affiliations:** 1 Department of Zoological Sciences, Kenyatta University, Nairobi, Kenya; 2 Center for Global Health Research, Kenya Medical Research Institute, Kisumu, Kenya; 3 Disease Control and Vector Biology Unit, London School of Hygiene and Tropical Medicine, London, United Kingdom; Arizona State University, United States of America

## Abstract

**Background:**

Mosquito Larval Source Management (LSM) could be a valuable additional tool for integrated malaria vector control especially in areas with focal transmission like the highlands of western Kenya if it were not for the need to target all potential habitats at frequent intervals. The ability to determine the productivity of malaria vectors from identified habitats might be used to target LSM only at productive ones.

**Methods:**

Each aquatic habitat within three highland sites in western Kenya was classified as natural swamp, cultivated swamp, river fringe, puddle, open drain or burrow pit. Three habitats of each type were selected in each site in order to study the weekly productivity of adult malaria vectors from February to May 2009 using a sweep-net and their habitat characteristics recorded.

**Results:**

All surveyed habitat types produced adult malaria vectors. Mean adult productivity of *Anopheles gambiae sensu lato* in puddles (1.8/m^2^) was 11–900 times higher than in the other habitat types. However, puddles were the most unstable habitats having water at 43% of all sampling occasions and accounted for 5% of all habitats mapped in the study areas whereas open drains accounted for 72%. Densities of anopheline late instars larvae significantly increased with the presence of a biofilm but decreased with increasing surface area or when water was flowing. Taking stability and frequency of the habitat into account, puddles were still the most productive habitat types for malaria vectors but closely followed by open drains.

**Conclusion:**

Even though productivity of *An. gambiae s.l.* was greatest in small and unstable habitats, estimation of their overall productivity in an area needs to consider the more stable habitats over time and their surface extension. Therefore, targeting only the highly productive habitats is unlikely to provide sufficient reduction in malaria vector densities.

## Introduction

The ecology and climate in many parts of the western highlands of Kenya supports stable transmission of malaria and increasing population pressure has led to changing land use practices, such as the clearance of natural swamps, massive deforestation and cultivation of crops in the valley bottoms [1]–[3]. These agricultural changes have created numerous water bodies exposed to the sun, providing ideal conditions for vector proliferation and increased malaria transmission [4], [5]. Malaria is caused by *Plasmodium* species, with *P. falciparum* being the most virulent human parasite in Africa. Parasites are transmitted from infected to uninfected people through infective bites by female *Anopheles* mosquitoes. In the western Kenya highlands, malaria is transmitted primarily by *Anopheles gambiae sensu stricto*, the most efficient vector within the *Anopheles gambiae* complex, as well as by *An. funestus* [1], [6]–[8]. The other species of this complex are *An. arabiensis*, *An. quadriannulatus*, *An. melas*, *An. merus* and *An. bwambae* [9], [10]. Efforts to control malaria primarily focus on the use of long-lasting insecticidal nets and prompt diagnosis and treatment [11], [12]. Additional indoor-residual spraying is prioritized for epidemic-prone areas at higher altitude. Nevertheless, due to the focal concentration of potential mosquito larval habitats in the valley bottoms [2], [13] larval source management (LSM) can provide a highly effective additional tool for vector control. A recent study showed that malaria incidence can be reduced by half in children protected by bednets and larviciding together compared to children only protected by bednets [14].

Traditionally LSM using larvicides is applied to all aquatic habitats in the target area [14]–[18]. However, not all aquatic habitats available at certain points in time contain mosquito larvae. Our recent study in the highlands of western Kenya indicates that at any time 38% (95% CI 30–56%) of habitats had anopheline early and 18% (95% CI 10–25%) had late instars larvae (Ndenga et al. unpublished data). Furthermore, the evaluation of LSM is based on the presence and/or density of larvae which does not accurately reflect that adult vectors would emerge from these habitats [19]. Very little is known about the relationship between the presence and density of immature mosquitoes and adult productivity of habitats. This is important since only habitats that produce adult vectors contribute to malaria transmission. If habitats that generate adult vectors could be identified, then LSM might be targeted at these sites [19], [20].

The term productivity has been conceived and used differently in malaria vector research to imply either presence/absence or density/abundance of anopheline larvae, pupae or emerged adults [3], [19], [21]–[25]. In this study, we define productivity as the number of adult mosquitoes emerging from one square meter of water with density of aquatic stages defined as abundance in one square metre. Mutuku and others [19] have suggested that pupal abundance depends on the habitat type. They showed for a small study area in the lowland of western Kenya that not all habitats were equally productive. In their study more stable burrow pits appeared to be the most productive habitats compared to more temporary habitats. To confirm whether such results can be more widely generalized, more research needs to be implemented in various environments to assess whether productivity per habitat type is similar or varies in different ecological settings.

This study was carried out in three sites within the western Kenya highlands to establish whether malaria vector productivity differed among six common habitat types during the long rainy season which is the main malaria transmission period. The specific objectives of this study were to determine whether different habitat types 1) differ in their larval and pupal abundance, 2) differ in their adult malaria vector productivity, and 3) whether habitat type or other confounding factors are associated with differences in abundance and productivity. The information presented in this paper is important as it can be used to determine whether LSM can be targeted only at the most productive habitat types or not.

## Materials and Methods

### Study site

**Figure 1:**
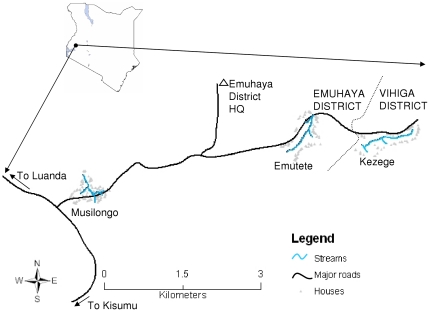
Study site map showing the three study areas: Musilongo, Emutete and Kezege.

This study was carried out within the western Kenya highlands in Western Province (Figure 1) from February to May in 2009. Sampling was done in Musilongo and Emutete areas of Emuhaya District and Kezege in Vihiga District. Emuhaya District was recreated from Vihiga District in 2007. Emuhaya District has an estimated human population of 270,000 people living on 173.2 km^2^ of land, while Vihiga District has 263,662 people living on 200.7 km^2^ of land (Emuhaya and Vihiga District Information Offices 2010). Human population density is high in these study areas which is estimated to be 0.1 persons per acre. Subsistence farming is the main economic activity in these districts with tea grown on small and scattered farms as the only cash crop. Other crops include maize, beans, bananas, vegetables, sweet potatoes, cassava, groundnuts, sorghum, sugar canes and napier grass. Quarrying for construction materials in the hilly and rocky areas and livestock keeping are also important economic activities that partly impact the creation of mosquito breeding sites. The average annual rainfall for this region is approximately 2,000 mm (based on data from 1960–1999) [8], [26], with long rainy seasons from April to June and short rainy seasons from October to November. The annual mean minimum temperature is 14°C and the maximum is 28°C, with the hottest months in January–February and the coolest months in July–August. Due to population pressure, natural swamps within valley bottoms in these three study areas are being reclaimed for crop cultivation by digging open water drains. Each of these study areas is characterized by a flat-bottomed valley with flowing streams and drains surrounded by hills. Musilongo (Universal Transverse Mercator (UTM) latitude 0.0208; longitude 34.6035; altitude 1500 meters above sea level (m.a.s.l.); area 0.16 km^2^); Emutete (latitude 0.0260; longitude 34.6358; altitude 1506 m.a.s.l.; area 0.24 km^2^) and Kezege (latitude 0.0264; longitude 34.6506; altitude 1545 m.a.s.l.; area 0.20 km^2^) are located along the Luanda – Majengo Road off Kisumu-Busia Road to the east.

### Mapping of study areas

Coordinate readings (latitude and longitude) and altitude of aquatic habitats, houses, major roads and streams were taken once using a Geographical Positioning Station (GPS) unit (GPS 12 XL, 15 metres accuracy, Garmin Ltd. 2003, Olathe, Kansas, USA). They were converted from decimal degrees to UTM and saved as dBASE IV (DBF4) type in Microsoft Excel 2003 then imported into ArcMap in ArcGIS (Environmental Systems Research Institute (ESRI) Redlands, California, USA) where they were projected into World Geodetic System (WGS)_84 UTM, Zone 36 N [26]. These maps were exported to Joint Photographic Experts Group (JPEG) image, then to Microsoft PowerPoint 2003 where final and additional information was added. Landmarks (major roads, paths and rivers) were used to determine the borders of each study area within a selected village.

### Selection of aquatic habitats

**Figure 2:**
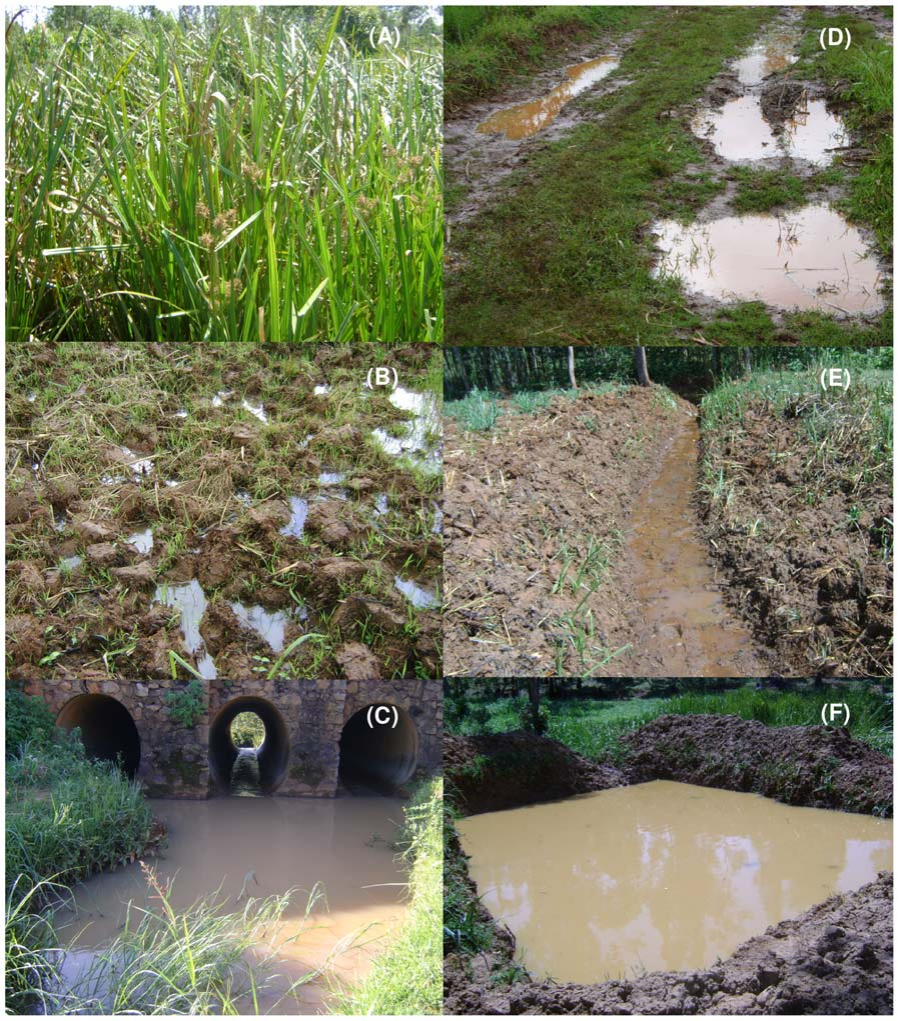
Habitat types sampled for mosquito aquatic stages: Natural swamp (A), Cultivated swamp (B), River fringe (C), Puddles (D), Open drain (E), and Burrow pit (F).

All aquatic habitats were mapped in the three study areas Musilongo, Emutete and Kezege from June 2008 to January 2009. These were sampled twice per month to determine the presence or absence of anopheline larvae. The number of times a given habitat had water during the sampling period was recorded and the proportion of time it had anopheline larvae determined. These habitats were grouped per habitat type as natural swamps, cultivated swamps, river fringes, puddles, open drains and burrow pits (Figure 2). These habitat types were described as follows: 1) natural swamps were water-logged sections of land with tall grasses (*Carex* species), reeds (*Phragmites australis*) and papyrus (*Cyperus papyrus*) which may not have been disturbed by human activities or reverted back to natural conditions after once being cultivated; 2) cultivated swamps were water-logged sections of land without tall grasses and where it was either being prepared for cultivation or was being cultivated; 3) river fringes were edges of a river or stream; 4) puddles were temporary collections of water in the valley bottoms that formed after rainfall; 5) open drains were open narrow drainages connecting to the main stream or disconnected ditches to lower water table and 6) burrow pits were excavations intentionally made by people to meet a specific purpose like fish-ponds, brick/sand-pits and grey-soil excavation pits dug in swampy areas for beautifying mud walls of houses. A total of 1,236 potential larval habitats were identified in the three study areas, 29 (2.3%) were natural swamps, 44 (3.6%) cultivated swamps, 10 (0.8%) river fringes, 67 (5.4%) puddles, 192 (15.5%) burrow pits and 894 (72.3) open drains. The selected habitats were randomly distributed in each of the study areas. Habitats were sampled weekly from 09:00h to 13:00h. Larval and pupae sampling begun at the end of the dry season in February 2009 and continued through the long rainy season ending at the end of May 2009, covering 17 consecutive weeks.

### Habitat characterization

The following information was collected and recorded for each habitat at the time of sampling: (1) date and week number of sampling; (2) study site name; (3) presence or absence of water; (4) length and width (cm) of water surface sampled; (5) water depth (cm, using an aluminum meter rule); (6) water flow (slow, fast or stagnant); (7) water temperature (°C, using an industrial thermometer); (8) presence and visually estimated coverage (percentage of surface area) of habitat with filamentous algae; (9) coverage (percent estimate) with emergent vegetation; and (10) coverage (percent estimate) with visible surface biofilm, which formed an oil-like layer on the water surface. Water surface area was calculated in square metres for every habitat at every sampling date.

### Sampling of immature mosquitoes and other aquatic organisms

A sweep net was used to determine the abundance of mosquito larvae and pupae and other aquatic macro-organisms per surface area. The sweep net (length = 40 cm, width = 15 cm, height = 30 cm) was made of a fine cloth that enabled the collection of newly hatched mosquito first instars larvae. A sweep net was preferred over the standard dipping method usually used in mosquito larval ecology studies because sweeping a defined surface area provides a measure per square meter of habitat. Furthermore, dipping underestimates the number of mosquito pupae and aquatic invertebrates [27], [28]. An area sampler was not used in this study because its use does not improve sampling success compared to dipping, hence not a reliable tool to estimate emergence of adult mosquitoes [25]. Robert and others [27] showed that sweep nets collected double the number of larvae and 10 times more pupae in fewer sweeps than dips. Sweep nets have been widely used to study changes in mosquito densities in breeding habitats and also their interactions with predators [29]–[31]. The net was inserted at an angle of 45° and gently dragged along the entire water surface of each habitat. Each habitat was swept at least three times until no more organisms were caught. In large habitats sweeping was carried out at the edges of the habitat where larvae and pupae aggregate [16]. Contents collected in the sweep net were emptied on a white tray to enhance visibility and counting of the sampled organisms. Immature mosquitoes were counted separately for early (1^st^ and 2^nd^) and late (3^rd^ and 4^th^) instars larvae of culicines and anophelines, and pupae (all species combined since they cannot be identified morphologically in the field). The number of invertebrates (odonata, coleoptera, heteroptera), fishes and tadpoles were also recorded. Early instars of anopheline and culicine larvae, late instars culicine larvae and other organisms were returned to the habitat, whereas late instars anopheline larvae and pupae were collected in 20 ml vials with a screw cork loosely tightened and half filled with water from the respective habitat and transported in a cooler box to the insectaries at KEMRI Kisumu, Kisian.

### Mosquito adult emergence

Field sampled mosquito larvae and pupae were kept in their respective water sample at ambient temperature and natural light in order to fully develop, pupate and/or emerge into adults. Emerged adults were morphologically identified into their respective genera and species [32], [33]. Individual specimens of female *An. gambiae s.l.* were further identified to species using the rDNA-polymerase chain reaction (PCR) method [34].

### Ethical considerations

Ethical approval for this study was granted by KEMRI/National Ethical Review Committee. Before any larval sampling was initiated, verbal consent to access compounds and farms was obtained from both administrative officials and residents during local village meetings in all study sites.

### Data analysis

Each of the six habitat types were sampled in three replicates per week in each of the three study sites, respectively, for 17 consecutive weeks. Each week a total of 54 samples were made (nine per habitat type) or 918 in 17 weeks (153 per habitat type). The abundance of aquatic organisms and productivity (emergence) of adults was calculated as the number of individuals per square meter per habitat per week. Generalized Estimating Equations (GEE) without intercept were used to calculate mean abundance and productivity of aquatic organisms, with habitat identity as subject units, habitat type as the factor and count data fitted to a negative binomial distribution with a log link function. Univariate General Linear Models (GLM) were applied to analyze statistical differences in ecological parameters among the six habitat types and Hochberg's GT2 post hoc test was used for comparisons between puddles and the 5 other habitat types. Proportions (habitat coverage with filamentous algae, emergent plants, biofilm and water stability) were arcsine transformed to normalize the data before they were analyzed. Univariate analyses were performed for study area, habitat type, water flow, water surface area, water temperature, emergent plant coverage and the presence of biofilm, filamentous algae, culicine larvae, invertebrates, tadpoles and fishes. Only factors found significant in univariate analyses to positively or negatively affect the abundance and productivity of mosquitoes in habitats were used for the final risk factor models. Analyses were performed using GEE on count data that were fitted a negative binomial distribution with a log link function to determine factors associated with the abundance of anopheline early and late instars larvae and the productivity of *An. gambiae s.l.*. Habitat identity was treated as the subject variable, week number as the within subject variable and an exchangeable correlation matrix chosen for the repeated measurements. Puddles were used as a reference habitat in all models because they had the highest abundance of mosquito larvae/pupae and productivity of adults. Productivity estimates of *An. gambiae s.l.* for each habitat type were obtained by multiplying averages of *An. gambiae s.l.* produced over the entire study period of 17 weeks per habitat type by the total number of habitat type in the study area and statistical differences among them determined using GEE. Missing data were excluded from the analysis. Analyses were performed with SPSS version 16.

## Results

### Habitat stability, larval colonization and their abundance

A total of 918 samples were made, 659 (71.8%) had water and 259 (28.2%) were dry. Stability of habitats varied among habitat types hence varying number of aquatic samples were made. River fringes were the most stable aquatic habitat type in the highland valleys that never dried (n = 153). All other habitat types had at least two to eight of their habitats dry at any given sampling visit between early February and late March when average weekly rainfall ranged from 0.62–34.4 mm. Puddles were the most unstable habitat type with habitats holding water on 43.1% of occasions (Table 1).

**Table 1:** Sample size, mean mosquito immature abundance and adult *Anopheles* productivity per m^2^ per habitat type (statistical comparisons made between puddles and other habitat types).

*Descriptive*	*Puddles*	*Natural swamps*	*Cultivated swamps*	*River fringes*	*Open drains*	*Burrow pits*
Number of habitat visits	153	153	153	153	153	153
Total number of wet samples taken	66	89	110	153	141	100
Habitat stability	43.1%(66/153)	58.2%(89/153)	71.9%(110/153)	100%(153/153)	92.2%(141/153)	65.4%(100/153)
Proportion of samples that contained *Anopheles* larvae	74.2%(49/66)	40.4%(36/89)	40.0%(44/110)	41.2%(63/153)	85.1%120/141)	74.0%(74/100)
Proportion of *Anopheles* positive samples that contained early instars only	38.8%(19/49)	52.8%(19/36)	68.2%(30/44)	76.2%(48/63)	48.3%(58/120)	55.4%(41/74)
Average proportion (95% CI) of late instars *Anopheles*/m^2^ in the *Anopheles* positive habitats	13.4%(7.6–19.2)	13.0%(6.1–20.0)	9.8%(3.6–15.9)	6.4%(3.2–9.6)	8.9%(6.6–11.2)	15.0%(9.1–20.5)
**Average immature abundance** †						
Anopheline early instars/m^2^	160.7(36.9–700.7)	4.4*(1.0–19.7)	10.0*(5.7–17.3)	0.3*(0.2–0.4)	8.5*(5.2–13.8)	6.4*(3.3–12.4)
Anopheline late instars/m^2^	11.6(3.2–42.6)	1.1*(0.2–6.9)	0.8*(0.3–2.0)	0.02*(0.01–0.04)	0.5*(0.3–0.8)	1.7*(0.5–5.4)
Culicine early instars/m^2^	116.1(21.6–622.8)	45.6(25.7–81.0)	17.2*(9.6–30.9)	0.1*(0.04–0.2)	0.7*(0.3–1.5)	2.3*(1.0–5.0)
Culicine late instars/m^2^	15.8(3.9–64.7)	6.3(3.1–12.5)	2.9*(1.3–6.3)	0.03*(0.01–0.1)	0.1*(0.05–0.2)	0.4*(0.2–1.3)
Mosquito pupae/m^2^	23.7(6.5–87.0)	6.7(1.9–24.0)	4.3*(2.3–8.0)	0.04*(0.02–0.1)	1.4*(0.4–5.5)	0.3*(0.2–0.6)
**Average ** ***Anopheles*** ** adult productivity** †						
*Anopheles gambiae s.l.*/m^2^	1.80(0.59–5.50)	0.06*(0.01–0.28)	0.16*(0.03–0.84)	0.002*(0.0005–0.01)	0.03*(0.01–0.08)	0.05*(0.03–0.09)
*Anopheles coustani*/m^2^	1.36(0.29–6.30)	0.52(0.08–3.36)	0.39(0.14–1.11)	0.001*(0.0003–0.004)	0.07*(0.03–0.15)	0.11*(0.03–0.37)
*Anopheles funestus*/m^2^	0.011(0.002–0.062)	0	0	0.001(0.0001–0.01)	0.017(0.0001–0.0040)	0
*Anopheles rhodesiensis*/m^2^	0.62(0.30–1.31)	0.05(0.01–0.29)	0.10(0.03–0.40)	0	0.02(0.01–0.05)	0.13(0.02–0.91)

*Indicate statistical significant difference with puddle at P≤0.05; Confidence Interval in parenthesis.

† Indicate analysis was done using GEE.

In all the 659 samples, 213 (32.3%) did not have any mosquito larvae whereas all the 147 (22.3%) samples that had culicine larvae also had anophelines. Anopheline larvae were found in 58.6% (386) of all samples, culicines in 31.4% (207) and mosquito pupae in 34.9% (230). Furthermore, late instars larvae which are frequently used as a proxy measure for habitat productivity [18], [35] were less sampled as anopheline late instars were found in 25.9% (171) of all samples whereas culicine late instars were found in 18.5% (122). In most cases, early instars larvae were identified in a sample (Table 1). Over three quarters of the samples from open drains, puddles and burrow pits contained anopheline larvae whereas more than half of all samples from natural and cultivated swamps and river fringes did not have any anophelines at all (Table 1). The abundance of early instars was higher in all habitat types compared to that of late instars. Of all *Anopheles* mosquitoes recorded per habitat and week, the average proportion of late instars was only 9.7% (5.6–14.3%) and was similar in all habitat types (Table 1). The mean abundance of anopheline early and late instars, culicine early and late instars and mosquito pupae per m^2^ varied between habitat types (Table 1). They were lowest in river fringes and highest in puddles as compared to the other habitat types. The mean number per m^2^ of anopheline and culicine larvae and their pupae were 26.9 (166.8/6.2), 10.1 (124.4/12.3) and 10.6 (22.9/2.2) times higher in puddles than in the other five habitat types pooled together, respectively.

### Habitat productivity

A total of 580 late instars anopheline larvae and 906 mosquito pupae, were collected and brought to the insectaries; 433 adults emerged; 239 (55.2%) anophelines, 165 (38.1%) *Culex* mosquitoes and 29 (6.7%) *Mansonia africana*. Of the emerged anopheline adults, 103 (43.1%) were *An. coustani*, 81 (33.9%) *An. gambiae s.l.*, 40 (16.7%) *An. rhodesiensis*, 14 (5.9%) *An. funestus* and 1 (0.4%) *An. squamosus*. Of the 81 *An. gambiae s.l.* mosquitoes, 45 were females which were further analyzed by PCR and 70.1% identified as *An. gambiae s.s.* and 29.9% as *An. arabiensis*. On average, highland habitats produced 0.91 (95% CI 0.31–2.78) adult anopheline mosquitoes per m^2^ of water surface. Approximately half of them (0.42 (95% CI 0.13–1.34)/m^2^) were malaria vectors (*An. gambiae s.l.* and *An. funestus*). The only anopheline species that emerged from all the six habitat types were *An. coustani* and *An. gambiae s.l.* Notably, *An. gambiae s.l.* adult mosquitoes emerged from 7.9% (52/659) of habitat samples. Nevertheless, productivity was not homogeneous for all habitat types (Table 1). Corresponding with the observations made on larval abundance, adult productivity was lowest in river fringes and highest in puddles. In puddles, on average 1.80 (0.59–5.50) adult *An. gambiae s.l.* emerged per m^2^. The difference in productivity was highly significant between puddles and other habitat types.

### Habitat characterization

Habitat characteristics for the different habitat types are summarized in Table 2. Generally, puddles shared many of the characteristics with other habitat types and none of them seemed to be specifically different for puddles. River fringes and burrow pits had significantly larger surface areas and deeper water levels compared to puddles but no differences were found between other habitat types. Average water temperature in puddles was significantly higher than in river fringes and open drains but similar to that in natural and cultivated swamps and burrow pits. Notably, invertebrate (water beetles, dragonfly and damselfly nymphs, water scorpions, backswimmers, creeping water bugs, and water striders) abundance was similar in puddles as in other larger habitat types, except in river fringes, and the highest fish (*Aplocheilichthys bukobanus*; maximum length 5.0 cm) abundance was found in burrow pits, puddles and river fringes. Tadpoles were most abundant in puddles and cultivated swamps. A visible biofilm covering the habitats was recorded in 52.4% (345/659) of all samples. Biofilm covered on average 14% (11–17%) of the water surfaces of puddles, open drains and burrow pits. Significant differences were found for river fringes, that rarely had any biofilm, and for natural and cultivated swamps, whose water surfaces were on average covered by half with biofilm. Filamentous algae were present in all habitat types except natural swamps. They covered on average only a small proportion of the water surface. All habitat types were to some extent covered with emergent vegetation. Puddles and river fringes had on average 10–12% of their surface area covered with emergent vegetation whereas in other habitat types these vegetations covered up to 41% of the surface area.

**Table 2:** Habitat characteristics per habitat type (statistical comparisons made between puddles and other habitat types).

*Descriptive*	*Puddles*	*Natural swamps*	*Cultivated swamps*	*River fringes*	*Open drains*	*Burrow pits*
**Habitat characteristics**						
Average surface coverage with biofilm (CI)‡	14%(10–21)	49%*(32–76)	47%*(34–64)	1%(0–2)	11%(5–25)	17%(7–42)
Average surface coverage with filamentous algae (CI)‡	2%(0–9)	0	3%(2–7)	2%(1–7)	8%*(5–15)	4%(2–7)
Average surface coverage with emergent vegetation (CI)‡	10%(5–22)	41%*(31–53)	29%*(21–40)	12%(8–18)	20%(12–31)	25%(14–43)
Average water depth in cm (CI)‡	7(5–11)	7(5–11)	6(3–11)	25*(17–36)	5(4–7)	19*(13–27)
Average water surface area in m^2^ (CI)‡	0.8(0.3–2.3)	2.8(1.7–4.6)	1.1(0.6–2.0)	9.7*(5.7–16.6)	3.5*(2.6–4.8)	4.7*(2.0–10.7)
Average water temperature in °C (CI)‡	22.8(21.4–24.4)	23.0(21.9–24.2)	22.7(21.7–23.8)	20.9*(20.3–21.5)	22.3*(21.1–23.6)	23.2(22.3–24.1)
Invertebrates/m^2^ †	4.5(1.1–19.0)	9.7(4.7–20.0)	12.2(7.8–19.3)	0.8*(0.5–1.2)	1.5(0.9–2.4)	3.5(2.0–6.3)
Fishes/m^2^ †	1.2(0.3–4.1)	0.03*(0.005–0.178)	0.01*(0.002–0.085)	0.4(0.2–0.6)	0.1*(0.04–0.24)	3.0(0.5–18.0)
Tadpoles/m^2^ †	68.7(20.4–231.3)	8.3*(2.5–27.6)	31.7(12.5–80.6)	0.7*(0.4–1.1)	1.7*(0.9–3.0)	4.3*(2.1–8.8)

* Indicate statistical significant difference with puddle at P≤0.05; Confidence Interval in parenthesis.

† Indicate analysis was done using GEE.

‡ Indicate analysis was done using GLM.

### Risk factor analyses for the presence of anopheline aquatic stages

Multivariate analyses were used to identify potential confounding factors that might be responsible for the significantly higher abundance of both early and late instars of anopheline larvae in puddles (Tables 3 and 4). Abundance of anopheline early instars larvae was positively associated with increasing water temperature, emergent plant coverage and the presence of fish but decreased with increasing water surface area, water flow and in the presence of filamentous algae (Table 3). Adjusting for these factors puddles did not differ from open drains in their probability of containing anopheline early instars larvae. Even though the presence of filamentous algae was significantly associated with early instars anopheline abundance in a univariate analysis this effect was no longer significant when other factors were considered. Similar to early anopheline instars, late instars abundance significantly decreased with increasing water surface area of the habitat and increasing water flow (Table 4). Increasing biofilm coverage of the habitat was associated with increasing anopheline late instars abundance (Table 4). Adjusting for these factors, the probability of increasing late instars anopheline abundance was similar in puddles and burrows pits. Emergent plant cover, filamentous algae and the presence of both fish and culicines were no longer significantly associated with late instars abundance in the multivariate analysis. Productivity of *An. gambiae s.l.* adult mosquitoes did not show any significant association with other habitat characteristics when the analyses were adjusted for habitat type.

**Table 3:** Factors associated with the abundance of anopheline early (1^st^ and 2^nd^) instars larvae.

*Parameter*	*Occasions (N)*	*Odds ratio*	*Lower CI*	*Upper CI*	*P*
**Water surface area (m^2^)**	659	0.858	0.804	0.916	<0.001
**Water Temperature (°C)**	659	1.132	1.042	1.229	0.003
**Emergent plant coverage (%)**	659	2.989	1.105	8.084	0.031
**Study area**					
Kezege	204	0.376	0.163	0.867	0.022
Emutete	227	0.653	0.269	1.585	0.346
Musilongo	228	1.000			
**Water Flow**					
fast	170	0.099	0.047	0.209	<0.001
slow	134	0.316	0.181	0.549	<0.001
stagnant	355	1.000			
**Filamentous algae**					
present	248	0.959	0.651	1.413	0.833
absent	411	1.000			
**Fishes**					
present	145	1.717	1.068	2.761	0.026
absent	514	1.000			
**Habitat type**					
burrow pit	100	0.088	0.019	0.414	0.002
open drain	141	0.312	0.071	1.373	0.123
river fringe	153	0.091	0.019	0.442	0.003
cultivated swamp	110	0.066	0.015	0.286	<0.001
natural swamp	89	0.030	0.004	0.206	<0.001
puddle	66	1.000			

CI = 95% Confidence Interval.

**Table 4:** Factors associated with the abundance of anopheline early (3^rd^ and 4^th^) instars larvae.

*Parameter*	*Occasions (N)*	*Odds ratio*	*Lower CI*	*Upper CI*	*P*
**Water surface area (m^2^)**	659	0.910	0.840	0.985	0.020
**Emergent plant coverage (%)**	659	0.677	0.197	2.323	0.535
**Biofilm coverage (%)**	659	3.355	1.132	9.943	0.029
**Water Flow**					
fast	170	0.268	0.121	0.595	0.001
slow	134	0.411	0.203	0.830	0.013
stagnant	355	1.000			
**Filamentous algae**					
present	248	0.858	0.541	1.361	0.515
absent	411	1.000			
**Fishes**					
present	145	0.888	0.539	1.462	0.640
absent	514	1.000			
**Culicines**					
present	206	1.403	0.642	3.068	0.396
absent	453	1.000			
**Habitat type**					
burrow pit	100	0.290	0.057	1.479	0.136
open drain	141	0.302	0.099	0.925	0.036
river fringe	153	0.039	0.010	0.160	<0.001
cultivated swamp	110	0.078	0.022	0.285	<0.001
natural swamp	89	0.088	0.014	0.552	0.010
puddle	66	1.000			

CI = 95% Confidence Interval.

### Estimated habitat type productivity

In order to identify habitat types that produce most vectors in a target area within a specified period of time, the mean number of adults emerging per m^2^ is not the only important factor. Other factors including, habitat stability and their total surface area need to be considered. During the 17 weeks sampling period, puddles were the least stable habitats compared to the others (Table 1). Therefore, the mean number of adults produced per m^2^ for the entire time period was 0.78 compared to 1.8 when only wet habitats were considered (Table 5). Assuming similar habitat stability we estimated how many adult *An. gambiae s.l.* were produced per m^2^ per habitat type in all three study sites over the study period (Table 5).

**Table 5:** Estimated overall productivity of adult *Anopheles gambiae s.l.* from the different habitat types during the study.

*Habitat types*	*Mean (CI) An. gambiae/m^2^ wet habitat*	*Mean (CI) An. gambiae produced during study*	*Total number of habitat types in the study area*	*Estimate of mean (CI) number of adult An. gambiae produced in study area/m^2^ during study*
Natural swamps	0.06*(0.01–0.28)	0.0354*(0.0083–0.1509)	29	1.0256*(0.2404–4.3758)
Cultivated swamps	0.16*(0.03–0.84)	0.1175(0.0219–0.6293)	44	5.1699(0.9653–27.6896)
River fringes	0.002*(0.0005–0.0122)	0.0025*(0.0005–0.0122)	10	0.0246*(0.0050–0.1221)
Open drains	0.03*(0.01–0.08)	0.0285*(0.0108–0.0757)	894	25.5170(9.6246–67.6516)
Burrow pits	0.05*(0.03–0.09)	0.0324*(0.0145–0.0726)	192	6.2218(2.7784–13.9326)
Puddles	1.80(0.59–5.50)	0.7821(0.2529–2.4187)	67	52.4010(16.9443–162.0528)

*Indicate statistical significant difference with puddle at P≤0.05; Confidence Interval in parenthesis.

## Discussion

Adults of the principal malaria vector in the western Kenya highlands, *An. gambiae s.l.*, emerged from all habitat types surveyed although significant variations in their productivity was recorded in which puddles had the most production and river fringes the least. Existence of such variations in the productivity of malaria vectors from habitats within the highlands of western Kenya greatly emphasizes the need to develop tools that can identify those habitats which could then be targeted during an LSM operation to make it more cost-effective [19], [20]. The productivity of *An. gambiae s.l.* adult mosquitoes reported here in puddles (1.80 mosquitoes/meter^2^/week) is comparable to that (1.82 mosquitoes/meter^2^/week) reported by Munga and others [3] when they used emergence traps in ditches and temporary pools located in farmlands from reclaimed swamps. In our study we see that puddles appear to be the most productive habitat type, but others [19], [36] have shown that they are less productive compared to burrow pits. It appears that this productivity may be very specific to certain environmental conditions and hence it can not be generalized. There is a challenge in distinguishing aquatic habitats using general definitions of habitat types. For instance, puddles can refer to a range of habitats which may include roadside water accumulations, tire tracks, hoof/foot-prints, rain and groundwater pools, and rock-pools. Conditions in these habitats can be very heterogeneous. In this study, puddles were small, shallow habitats that were exclusively formed in valley bottoms by rain and high ground water level. These puddles were often associated or in close vicinity with overflowing rivers, streams, drains or swamps, hence fishes were frequently found in them. Having a very clear description of a specific habitat type is crucial especially when field teams may be expected to identify and treat certain type(s) and not others. The above example shows that what is defined as puddle in one area might be very different from a puddle in another area and productivity data might not be generalized from one area to another. Therefore, in every target area all habitat types would need to be categorized and field teams trained on how to identify the habitat types and their individual productivity established for local malaria vectors. Therefore, using habitat type to target LSM could only work if and after such a detailed preliminary survey has been undertaken.

Availability and stability of aquatic habitats are very crucial in determining year round productivity of malaria vectors [13]. Although in the highland valleys smaller and more temporary habitats have been identified to produce the highest number of adult malaria vectors per m^2^, their low stability over time and their overall small surface area greatly reduce their productivity. This is when compared to the less productive but stable habitats with greater surface areas like burrow pit and open drains. Estimated productivity of *An. gambiae s.l.* adults still identified puddles as the most productive habitats, followed by the open drains because of their large number and persistence. Considering that their surface area is probably much higher than those of puddles, open drains might overall contribute similar if not more to all vectors emerging in the target areas. However, since it has been shown that most malaria transmission in Kenya occur 1–2 months after the peak in rainfall in May, June and July [37], [38], these temporary habitats should not be underestimated within the highland areas of western Kenya and would need to be included into any targeted approach for larval control. Nevertheless, targeting puddles alone will probably not reduce the vector burden to such an extent that reduction of malaria prevalence is achieved.

The finding that puddles shared many of ecological characteristics with the other habitat types and that none of them was unique for this habitat type implies that the parameters measured in this study may not be used by field teams to identify the most productive habitat types for the adults of *An. gambiae s.l.* and thus target treatment during an LSM operation in this area. However, some of these parameters played significant roles in either enhancing or diminishing the development of *Anopheles* mosquitoes in their aquatic stages, with a possibility of indirect effects on their productivity of adult mosquitoes. Abundance of anopheline larvae increased with increase in water temperature and in the coverage of both emergent plants and biofilm in habitats. All the six habitat types had their water temperatures within the optimal range of 16 to 34°C that allows larvae of *An. gambiae s.s.* to survive and develop into adults [39]. However, higher temperatures create conditions more favourable for the survival of *An. gambiae* larvae [3], [19]. The average air and water temperatures are far lower in highland areas than in lowland areas [40], [41]; therefore site selection of the gravid female and larval survival might be affected by temperature. Smaller habitats tend to experience greater day/night fluctuations in temperature than larger ones, which might affect their productivity in highland areas. Presence of emergent plants in aquatic habitats has been positively, negatively or not associated with that of *Anopheles* mosquitoes in different places [22], [25], [42]–[45]. This might be because the presence of emergent plants in habitats may be describing a wide variety of plants at varying coverage levels, hence conflicting results are obtained. It is interesting to note that the presence of invertebrate and vertebrate predators was not significantly associated with low larval abundance; frequently fish and invertebrates co-existed with mosquito larvae in habitats. Fillinger and others [25] obtained similar results in The Gambia. The positive and significant association between the presence of biofilm and the abundance of anopheline late instars larvae observed here could indicate that the complex organic matter of bacteria and algae contained in biofilms provide important food sources for larvae which possibly other habitats in the highland areas might be deprived of [46]. However, there is need to do more work on the biofilms found in these highlands in order to establish, characterize and quantify their contents and specifically the suggested mosquito food sources. This observation contradicts other studies where oily layers on water have been associated with decreased survival due to the blockage of larval siphons [47], [48]. This might be due to a different composition of the layers or due to extension over the water surface. Notably, biofilms in this study only covered a small proportion of the aquatic habitats. Abundance of anopheline larvae decreased with increase in water surface area and its flow rates; this is an expected dilution effect. The finding that all the samples that had culicine larvae also had anopheline larvae implies that the presence of culicine larvae in habitats in the highlands can be used as an indicator of the presence of anopheline larvae, but not vice versa. Lack of correlations in the presence of both these species in samples is contrary to the findings obtained in the lowlands that these species coexist in the majority of the habitats [49]. This was because many additional samples contained only the anopheline larvae, an indication that culicines are not dominant species in habitats within the highlands. It is worthy noting that the selectiveness exhibited by ovipositing gravid mosquitoes [50]–[52] may to some extent determine the final productivity of aquatic habitats within these habitat types.

Previous studies on indoor resting mosquitoes reported *An. gambiae s.s.* to be the only sibling species of the *Anopheles gambiae* complex in the highlands of western Kenya [1], [7], [8]. In 2006, Munga and others [3] reported 1.3% occurrences of *An. arabiensis* from adults collected in emergence traps. In 2009, Fillinger and others [14] have reported a 3% occurrence of *An. arabiensis* from indoor resting data. In this study, we report that about a third of all *An. gambiae s.l.* collected directly from habitat types are *An. arabiensis*. This is an indication of an increase in the occurrence of *An. arabiensis* within these high altitude areas, implying that this species is slowly establishing itself in this highland. There are at least three possible explanations for this phenomenon. The promotion of long lasting insecticide treated nets (LLINs) in this area may have greatly reduced numbers of the endophilic malaria vector species which has resulted into increased proportions of the exophilic *An. arabiensis* as reported in other areas [53]–[55]. The initial failure to get *An. arabiensis* in the highlands may have been due to sampling bias with few samples made outdoors where *An. arabiensis* prefers to rest and feed [56]. Since *An. arabiensis* generally prefers drier areas [57], [58], then increased temperature and reduced relative humidity as a result of deforestation and changes in climate may have contributed to the establishment of this species within the highlands regions [59], [60]. This colonization may have been enhanced by the fact that *An. arabiensis* is abundant in the areas surrounding the western Kenya highland region [1].

Two limitations were identified in the course of carrying out this study. Transferring late anopheline larvae and all mosquito pupae from their natural habitats to the insectaries isolated them from their predators which could potentially have reduced adult productivity. This may have caused us to slightly overestimate emergence. Emergence rates from collected larvae and pupae were very low which might represent natural mortality [19], [61], [62], however, we can not exclude that the transport from the field site to the laboratory might have increased mortality rates. In this case we may have underestimated their emergence. The sampling efficiency of sweep nets is likely to have been greater in small and shallow bodies of water than wider and deeper ones as they could not have escaped in small habitats, which might be partly responsible for higher abundance of larvae in smaller habitats like puddles.

In conclusion, the findings from this study and from published work show that the productivity of malaria vectors from different habitat types are highly heterogeneous. Here, all habitat types produced adult malaria vectors. The overall productivity of *An. gambiae s.l.* adults from habitats in a given study area depends on the production per square meter of each habitat type, the stability of the habitat over time and the water surface extension of the habitat type. Even though some habitat types produce larger numbers of adult malaria vectors per water surface area than others, our results do not indicate that targeting for example puddles alone in the highland areas would provide sufficient reduction in vector densities. Fillinger and others [25] have made a similar conclusion that targeting LSM might be impossible in other environments like The Gambia. Therefore, we recommend the traditional method of applying larvicides in all aquatic habitats be continued within the western Kenya highlands instead of using habitat type as a determine factor.
